# Efficacy of a preoperative smoking cessation intervention in orthopedic and general and urological surgery patients: A study protocol for a randomized clinical trial

**DOI:** 10.18332/tid/203550

**Published:** 2025-10-17

**Authors:** Eva Gavilan, Esteve Fernández, Joan Minguell, Enrique Trilla, Josep M. Sánchez, Eloy Espín-Basany, Esperanza Zuriguel, Consuelo Álvarez, Irene Montllor, Miquel Ferré, Silvia Aneas, Agustín Gayubas, Cesar Botana, Marta Colmenero, Gemma Pérez, Natalia Rodríguez, Nuria Gili, Cristina Martínez

**Affiliations:** 1 Surgical Area, Vall d’Hebron University Hospital, Barcelona, Spain; 2 Escuelas Universitarias Gimbernat (EUG), adscrita a la Universitat Autonoma de Barcelona, Barcelona, Spain; 3 International University of Catalonia, Barcelona, Spain; 4 Multidisciplinary Nursing Research Group, Vall d’Hebron Research Institute, Barcelona, Spain; 5 Secretariat of Public Health, Department of Health, Government of Catalonia, Barcelona, Spain; 6 Tobacco Control Unit, WHO Collaborating Centre for Tobacco Control, Institut Catala d’Oncologia, Barcelona, Spain; 7 Tobacco Control Research Group, Institut d’Investigacio Biomedica de Bellvitge, Barcelona, Spain; 8 Faculty of Medicine and Health Sciences, University of Barcelona, Barcelona, Spain; 9 Centro de Investigacion Biomedica en Red de Enfermedades Respiratorias (CIBERES), Madrid, Spain; 10 Orthopedic Surgery and Traumatology Department, Vall d’Hebron University Hospital, Barcelona, Spain; 11 Department of Medicine, Autonomous University of Barcelona, Barcelona, Spain; 12 Reconstructive Surgery of the Locomotor System (CRAL), Vall d’Hebron Research Institute, Barcelona, Spain; 13 Urology Department, Vall d’Hebron University Hospital, Barcelona, Spain; 14 Kidney Physiopathology Research Group, Vall d’Hebron Research Institute, Barcelona, Spain; 15 Preventive Medicine Department, Vall d’Hebron University Hospital, Barcelona, Spain; 16 Colorectal Surgery Unit, General Surgery Department, Vall d’Hebron University Hospital, Barcelona, Spain; 17 Management of Knowledge and Evaluation, Vall d’Hebron University Hospital, Barcelona, Spain; 18 Traumatology Department, Vall d’Hebron University Hospital, Barcelona, Spain; 19 Presurgical Αrea, Vall d’Hebron University Hospital, Barcelona, Spain; 20 Anesthesiology Service, Vall d’Hebron University Hospital, Barcelona, Spain; 21 Department of Public Health Nursing, Mental Health, and Maternal-Infant Care, School of Nursing, L’Hospitalet de Llobregat, University of Barcelona, Barcelona, Spain; 22 Philip R. Lee Institute for Health Policy Studies, University of California San Francisco, San Francisco, United States

**Keywords:** smoking cessation, presurgical intervention, surgical complications

## Abstract

Tobacco use is a major risk factor for any surgical intervention. Offering patients help with giving up smoking before surgery increases cessation rates and lowers the risk of complications. The aim of this clinical trial is to evaluate the efficacy of an intensive presurgical intervention for promoting smoking cessation in smokers undergoing either orthopedic surgery with implants or general/urological surgery. We will conduct a stratified randomized clinical trial [intervention group (IG) and control group (CG), 1:1 allocation] at the Vall d’Hebron University Hospital, Barcelona, Spain. The IG will receive intensive help to quit smoking (psychoeducational and behavioral support, nicotine replacement therapy with follow-up, and an information leaflet). The CG will receive brief advice and the same information leaflet in a single session. Sample size was calculated to include four equal groups (IG and CG in both types of surgery) with an estimated difference of 15 points in abstinence between IG and CG; assuming a loss to follow-up of 10%, a total of 232 subjects will be needed (58 per group). The primary dependent variables are self-reported and verified abstinence from tobacco consumption (expired CO) and surgical complications. We will conduct descriptive and bivariate statistical analysis for independent data. Logistic regression will be performed to assess the efficacy of the intervention. The relative risk of surgical complications will be calculated using Cox regression models. Patient recruitment began in May 2023. This trial will be the first to evaluate an intervention of this nature in Spain. If its efficacy is demonstrated, the results will support the design of a protocol for a smoking cessation program aimed at smokers who are scheduled for surgery.

**CLINICAL TRIAL REGISTRATION:**

The study is registered on the official website of ClinicalTrials.gov

**IDENTIFIER:**

ID NCT 05961813

## INTRODUCTION

The global tobacco epidemic still causes 8 million deaths each year^1^. Smoking is a risk factor for premature death and disability^2^, and is a major risk factor in all kinds of surgery^3^. Smoking is associated with high mortality^4^ and an increased risk of general morbidity^5^ which may lengthen hospital stay, raise readmission rates and ultimately increase healthcare costs^6^.

It has been shown that tobacco consumption poses a particularly significant risk in several surgical specialties^7^. Specifically, in colorectal surgery, smokers have a 30% higher risk of mortality and morbidity after colorectal resection than people who have never smoked or are ex-smokers^8^. In urinary tract surgery, the risk of post-surgical complications after radical cystectomy is almost twice as high in smokers, particularly wound dehiscence and myocardial infarction^9^, and after radical prostatectomy smokers present a higher risk of pneumonia and unplanned intubation requirement^10^. In the field of orthopedic surgery, smokers have a higher than average rate of bone mineral loss, and their fractures take longer to heal^11^. One systematic review concluded that smoking significantly increases periprosthetic intra-articular space infection and surgical wound complications in primary hip and knee joint replacement surgeries^12^.

Quitting smoking before surgery reduces surgical complications and provides benefits in the short-, medium- and long-term^13^. Clinical trials have shown that offering help to stop smoking before surgery encourages smoking cessation, reduces the risk of cardiovascular complications and surgical wound infection, and helps to shorten hospital stay^14^.

Most smoking treatment guides, including the one published by the US Public Health Services^15^, recommend the 5 As brief intervention model, which comprises five steps or actions: 1) Ask, 2) Advise, 3) Analyze, 4) Assist, and 5) Agree on follow-up. In addition, intensive interventions should be incorporated that provide psycho-educational/behavioral support, pharmacological therapy^15^ and follow-up visits for approximately one year to prevent relapses^16^. In the context of surgery, previous studies have shown that multi-component intensive interventions with sessions starting before surgery and followed up over time achieve the highest rates of abstinence^14,17^.

According to the Spanish Ministry of Health’s Survey on Alcohol and Drugs, 33.1% of the country’s population aged ≥15 years still smokes^18^. Smokers are likely to suffer from chronic and acute tobacco-related pathologies, and generate high economic and social costs for the country’s health system^19^. Many will need surgery to treat medical conditions that may or may not derive from their tobacco use.

The need to undergo surgery is associated with a higher probability of successful smoking cessation^20^. But in spite of the opportunity that imminent surgery represents for giving up smoking, tobacco consumption in the pre-surgical period is not an issue that is systematically addressed13. In Catalonia, the identification of smokers who are motivated to give up (and the provision of support for their efforts to do so) does not reach the desired levels^21^. It is clear that effective interventions should be planned at key times such as pre-surgical visits.

The World Health Organization (WHO) urges countries to include smoking cessation programs for pre-surgical patients^22^ as a measure to reduce complications and improve long-term health. In Spain, however, there is no systematic pre-surgical intervention for cessation of tobacco use, nor has any such intervention been evaluated to date. According to the WHO’s ‘Research on the Implementation of Health Policies: a Practical Guide’^23^, applied research is needed to evaluate the effectiveness of interventions, taking into account the real situations of healthcare systems and patients. Conducting studies that demonstrate not just the effectiveness of interventions but also the feasibility of their introduction into real healthcare systems favors their subsequent implementation, sustainability, and dissemination.

The aim of the trial described here is to evaluate the efficacy of an intensive pre-surgical intervention to promote smoking cessation in smokers scheduled to undergo either elective orthopedic surgery with implants or general/urological surgery. These types of surgeries provide sufficient time for preoperative planning and the implementation of a smoking cessation intervention (4 weeks), enabling us to compare the impact across various surgical types and highlight potential differences in outcomes.

The primary outcome is tobacco use (Yes/No, defined as active use in the last 7 days) and the secondary outcome is the presence of surgical complications (classified into respiratory, cardiovascular, wound complications, and mortality).

The specific objectives are the following:

1. To compare the effectiveness of the intervention in terms of abstinence, patient evolution, motivation and the attempts to quit smoking between the patients in the IG and the CG undergoing one of the two types of surgery.
2. To compare the length of hospital stay (in days) and the number of re-admissions between the IG and the CG according to type of surgery.
3. To compare surgical and post-surgical complications and mortality rates between the IG and the CG up to 90 days post-surgery.
4. To assess whether the intervention can be implemented in the health system in Spain.

These objectives will be analyzed by comparing the results obtained in the IG and CG at the following time points: before surgery (at the beginning of the study), on the day of surgery, and at 1, 3, 6 and 12 months post-surgery (Table 1).

**Table 1:** Plans for assessment and collection of different variables in surgical patients who participate in the RCT at the Vall d´Hebron University Hospital, Barcelona (N=232)

*Variables*	*Baseline*	*Day of* *surgery*	*1 month* *post-surgery*	*3 months* *post-surgery*	*6 months* *post-surgery*	*12 months* *post-surgery*	*Re-admission*
Sociodemographic data	x						
Specialty	x						
Type of SI	x						
Smoker (yes/no)	x	x	x				
Type of consumption	x						
Amount of consumption	x	x	x (if s)	x (if s)	x (if s)	x (if s)	
Fagerström test	x		x (if s)	x (if s)	x (if s)	x (if s)	
Stages of change	x		x (if s)	x (if s)	x (if s)	x (if s)	
Richmond test	x		x (if s)	x (if s)	x (if s)	x (if s)	
Previous abstinence	x						
Previous drugs	x						
Living with smoker	x						
Comorbidities	x						
General self-efficacy scale	x						
CO-oximetry		x					
ASA PS classification		x					
ATB prophylaxis		x					
Duration of surgery		x					
Blood transfusion		x					
SatO2 at start of surgery		x					
Complications of surgery		x					
Complications at time of recovery room care discharge after surgery		x					
Attempts to quit			x (if s)	x (if s)	x (if s)	x (if s)	
Decrease in consumption			x (if s)	x (if s)	x (if s)	x (if s)	
Pharmacological treatment			x	x	x	x	
Is lack of NRT funding a problem?			x	x	x	x	
Readmission (yes/no)							x
Reason for readmission							x
SSI							x
Postoperative mortality							x

RCT: randomized clinical trial. SI: surgical intervention. ASA PS: American Society of Anesthesiologists physical status scale. ATB: antibiotic. SatO2: oxygen saturation. NRT: nicotine replacement therapy. SSI: surgical site infection. if s: if smokes.

## METHODS

The SPIRIT 2013 RCT protocol reporting guide was used^24^ (see Supplementary file), this study is a stratified randomized clinical trial (RCT) with two intervention groups (IG) and two control groups (CG) (n=58 per group) of smokers scheduled to undergo either orthopedic surgery with implants or general/urological surgery. Subjects will be followed up to 12 months after surgery.

### Study population

The study population will be adult patients scheduled to undergo the following surgeries at the Orthopedic Surgery and Traumatology, General Surgery and Urology services at the Vall d’Hebron University Hospital in Barcelona: knee replacement, hip replacement, instrumented spinal arthrodesis, anterior cruciate ligament repair, colorectal surgery for colon and rectal neoplasia, cystectomy, radical prostatectomy and partial or radical nephrectomy. The inclusion criteria are: adults aged ≥18 years; conscious and oriented in space, time and person; scheduled for surgery at least four weeks after inclusion in the surgical schedule; daily and occasional smokers (occasional: do not smoke every day); resident in the healthcare area and have no plans to change address in 12 months. The exclusion criteria are: pregnancy; smokers already undergoing treatment to stop smoking; inability to give informed consent due to memory loss or insufficient understanding of Spanish or Catalan; non-primary nature of knee or hip replacement; scheduled to participate (or already participating) in a clinical trial in which nicotine replacement therapy (NRT) is an exclusion criterion.

### Sample size and randomization

Every year, approximately 1800 surgical procedures of these kinds are performed at our hospital, of which approximately 25% are carried out in smokers. The sample size has been calculated assuming four equal groups with an estimated difference of 15 percentage points between the predicted rates of abstinence at twelve months in the IG (20%) and the CG (5%). With an expected loss to follow-up rate of 10%, 232 subjects will be needed (58 per group). The sample size has been calculated using the software STATA (StataCorp. 2023. *Stata Statistical Software: Release 18*. College Station, TX: StataCorp LLC). Patients will be assigned to their group by computer-generated random sequence (Stata *ralloc* function) (1:1 assignment) (REDCap). The study will be conducted in a single-blind manner (blinded for surgeons, collaborating researchers, and outcome analysts). There are no circumstances that would necessitate unblinding.

### Outcome variables

The main outcome variable will be tobacco use (Yes/No, defined as active use in the last 7 days)^25^ at the various time points. Abstinence validation will be performed with CO-oximetry on the day of surgery and at 12 months for subjects who declare themselves abstinent (patients with CO >6 ppm will be considered as active smokers)^26^. At the other time points (start of the study, and at 1, 3, 6 and 12 months) abstinence will be self-reported by the patient. The following secondary variables will be recorded at each assessment: 1) type of tobacco product (cigarettes, rolling tobacco, cigars, etc.); 2) pattern of consumption of each type of tobacco product (daily or occasional, and daily/weekly amount); and 3) level of nicotine dependence, using the Fagerström test (low 0–3, medium 4–6, and high 7–10)^27^. Other variables that will be assessed include motivation and self-efficacy, willingness to quit smoking according to the Prochaska and Di Clemente model^28^, level of motivation using the Richmond test^29^; and the General self-efficacy scale (based on Bandura’s social cognitive theory)^30^.

Another outcome variable will be surgical complications classified into the following groups and assessed on the day of surgery, on discharge from recovery-room care, hospital discharge, and up to 90 days post-surgery: 1) respiratory complications (bronchospasm, respiratory failure requiring oxygen and active treatment, respiratory superinfection requiring antimicrobial treatment, pleural effusion, other); 2) cardiovascular complications (deep venous and pulmonary thromboembolic disease, hypertensive crises, angina, new onset arrhythmias/blockages, acute myocardial infarction, heart failure, other); 3) postoperative complications of the surgical wound (dehiscence, fistula or infection of the internal or deep wound, seroma, wall abscess or fistula of the external wound with antimicrobial treatment and positive cultures, other); and 4) mortality (during hospitalization and within 90 days of surgery). All complications after the day of surgery will be recorded taking into account data from the patient’s medical history. Variables related to surgery: surgical time, blood transfusions (to be assessed as possible confounding variables for post-surgical complications and mortality). Variables related to hospital care: length of hospital admission (in days); and readmissions required within 12 months of hospital discharge.

### Independent variables

The main independent variable will be ‘implementation of an intensive pre-surgical intervention to stop smoking’ (Yes/No). In addition, the following data will be recorded:

1. Sociodemographic variables such as sex, age, marital status, living with a smoker, education level, type of surgery.
2. Comorbidities.
3. Pharmacological treatment: none/NRT according to the recommendations of the different guidelines^15,31^.

The variables to be recorded and the frequency of recording are detailed in Table 1.

### Procedure

This pragmatic intervention will be carried out at different hospital services and will involve healthcare professionals from different ambits for evaluation. The intervention will begin in the pre-surgical period, at the pre-anesthetic visit, where smokers who meet the inclusion criteria outlined in the protocol will be invited to participate. Those who agree will receive more detailed explanations of the nature of the study and they will be asked to provide written informed consent. An information leaflet (see Supplementary file) will be provided to all patients who agree to take part. Within 24–48 hours they will receive a telephone call during which baseline data will be recorded, and at the end of the interview, patients will be randomly assigned to the IG or the CG. Participants will receive the following, according to their group.

*Intervention group (IG)*

Intensive intervention consisting of two pre-surgical visits starting at least four weeks before surgery (depending on the type of surgery) + an information leaflet (see Supplementary file) + pharmacological therapy (NRT if needed) + four follow-up consultations by telephone, carried out by expert staff trained in intensive smoking interventions (at 1, 3, 6 and 12 months after surgery) with the aim of maintaining abstinence.

*Control group*

A single visit with brief advice to stop smoking and the same information leaflet between three and six weeks before surgery (depending on the type of surgery) provided by healthcare personnel. Follow-up after discharge will be by telephone at 1, 3, 6 and 12 months after discharge, but no more advice will be given.

**Figure 1:**
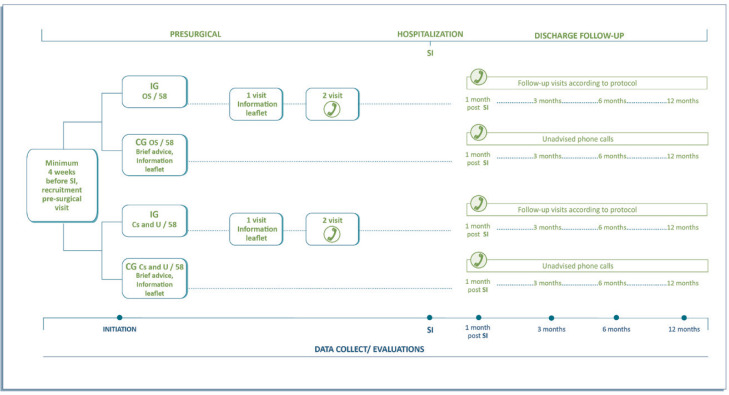
Intervention procedure in the RCT at the Vall d’Hebron University Hospital, Barcelona (N=232)

The intervention procedure is detailed in Figure 1.

### Components of the intensive intervention

The intervention is based on the recommendations of smoking cessation treatment guidelines^15,31.^ The intervention aims to ensure that the patient stops smoking before surgery and remains abstinent for as long as possible. It comprises an initial face-to-face visit lasting between 30 to 45 minutes approximately four weeks prior to surgery, and a second telephone visit approximately two weeks prior to surgery. After surgery, patients will be followed up by telephone, with visits after 1, 3, 6 and 12 months. The professionals who will carry out the intervention are experts in the treatment of tobacco addiction and are members of the Preventive Medicine service of the hospital. The components of the various visits are detailed in Table 2.

**Table 2:** Components of intensive intervention visits in the RCT at the Vall d’Hebron University Hospital, Barcelona (N=232)

*Visits*	*Components*
**First visit** Face-to-face	Assessment of nicotine dependence (Fagerström test). Assessment of stage of change (Prochaska and DiClemente). Assessment of motivation (Richmond test). Assessment of general self-efficacy. Establishment of a plan to quit smoking, emphasizing the benefits of the upcoming surgery. If possible, setting a date to quit, ‘D’ day (try to make it as soon as possible before surgery). Prediction of difficulties that may arise, particularly the first few weeks, including symptoms of nicotine withdrawal syndrome. Recommendation of the use of pharmacological therapy (NRT) if necessary, except when contraindicated. Offer practical advice for problem solving/skills training, learning cognitive and behavioral activities to cope with the desire to smoke. Discussion of difficulties/triggers and how the patient can overcome them (avoiding triggers, changing routines). Description of previous experiences, discussion of what helped and what did not, build on success. Facilitate clinical support throughout the process. Agree on follow-up.
**Second visit** By phone prior to surgery	Assessment of self-reported abstinence. Reinforcement of abstinence before surgery. Assessment of difficulties and problems. Reinforcement of behavioral coping skills and social support. Assessment of adherence to drug treatment and any necessary changes. Recommendation of relapse prevention strategies for patients who remain abstinent. Offer of congratulations emphasizing the benefits of upcoming surgery. Arrangement of follow-up.
**Follow-up visits** By phone after surgery	Assessment of self-reported abstinence. Assessment of treatment adherence and difficulties in following it. Reinforcement of behavioral strategies to cope with smoking urges. Relapse prevention. Recording data on tobacco use and variables if the patient continues to smoke. Measurement of CO-oximetry at the end of the study. Prompt notification of patients who do not attend, so as to try to arrange visits as soon as possible.

### Data collection

Four instruments have been created to record the variables to be studied at each assessment, and another one for randomization using the REDCap application. All the study researchers will be assigned a personal username and password that they will use to access the data and will only be able to access the instruments for which they have authorization. Patients will be informed about the use of the data and the person responsible for their treatment, as well as their rights of access, rectification, cancellation and opposition. Approximately 48 hours after signing the informed consent, baseline data will be recorded and randomization performed. Once the study has finished, the researchers will no longer have access to the database. The Vall d’Hebron Research Institute (VHIR) will be responsible for properly storing the data until their destruction. The database will not identify any of the participants since it will contain only individual encrypted codes. The intervention and data collection procedures using the REDCap application will be previously tested to assess their suitability, if necessary.

### Data analysis

The implementation of the intervention will be evaluated with data on fidelity, dose and scope with respect to the protocol (time spent on visits, use of instruments, barriers and problems detected, recruitment of participants, adherence to treatment, satisfaction). This stage will be carried out prior to the analysis of the results of the RCT. The efficacy of the intervention in terms of abstinence and post-surgical complications will be evaluated by comparing IG and CG. The data will be analyzed separately in the two surgery groups (orthopedic with implants and general/urological). Using the data obtained at the beginning of the study (i.e. a questionnaire with baseline data on tobacco consumption and sociodemographic variables) and the data from the different assessments over the course of the study, descriptive analyses will be performed and a contingency table with bivariate analysis will be created. Logistic regression will be performed to assess the efficacy of the intervention and the rest of the outcome variables. As part of our secondary analyses, we will explore whether the intervention effect varies across different surgical groups by including an interaction term (type of surgery × intervention) in our regression models. Considering the longitudinal nature of our data, a generalized linear mixed binomial family model to account for the correlation between repeated measurements within individuals will be used to assess the evolution of smoking cessation. Hazard ratios (HRs) of surgical complications in the IG and the CG will be calculated using Cox regression models. The proportional hazards assumption will be evaluated using statistical tests (e.g. Schoenfeld residuals) to ensure the validity of the Cox model. The number of patients needed to be treated to achieve cessation in one patient (NNT, and its 95% confidence interval) will be calculated over the one-year period. The NNT is the inverse of the attributable risk and is useful for conveying the efficacy of the intervention to patients, managers and clinicians. All analyses will be performed on an intention-to-treat basis. The level of statistical significance will be set at α=0.05 and the Bonferroni correction will be considered to adjust for multiple comparisons.

### Ethical considerations

The protocol for this intervention has been approved by the Vall d’Hebron University Hospital’s Drug Research and Ethics Committee [reference: PR(AG)367/2018, v2]. The study protocol has been registered at ClinicalTrials.gov, (2023) (NCT05961813). The study will be conducted in accordance with the Biomedical Research Act 14/2007 and the principles established by the World Medical Association’s 2013 Declaration of Helsinki and its subsequent amendments. The processing, communication and transfer of participants’ personal data will comply with EU Regulation 2016/679 of 27 April 2016 on Data Protection and Spanish Organic Law 3/2018 of 5 December on the protection of personal data and the guarantee of digital rights. Informed consent will be requested from all participants.

## DISCUSSION

Smoking is a major risk for patients undergoing surgery. Surgical complications are associated with smoking increasing readmission rates, prolonging hospital stays and ultimately increasing healthcare costs.

Smokers scheduled for surgery are at an ideal time to try a quit attempt^32^. Intensive interventions initiated at preoperative visits are known to be effective^17^ and can reduce complications. Despite this evidence, little help in this regard is offered during pre-surgical visits^33^. This study is the first in Spain to evaluate the effectiveness of an intensive pre-surgical intervention comprising psychological and behavioral support, pharmacological therapy, and a one-year follow-up program designed to help patients quit smoking before they undergo surgery.

The sample size and stratification will allow us to perform subgroup analyses, and the one-year follow-up period will enable us to obtain longterm abstinence data comparing CO-oximetry measurements made on the day of surgery and at the end of the study.

The main expected result is the achievement of an abstinence rate that is 15% higher among patients in the IG compared to those in the CG, and, at the same time, a reduction in the risk of complications in the IG. The analyses will also consider sociodemographic variables, comorbidities, type of surgery, and surgical variables such as duration and transfusions required. Other important results for comparison between groups will be motivation, dependence, cessation attempts, and reduction in consumption.

### Limitations

The main limitation of the study is the likely loss of subjects; however, this issue has been taken into account in the calculation of the sample size and in the inclusion criteria. In addition to the limitation of single blinding, we have identified other potential limitations in our study, such as the possibility of selection bias due to the non-random nature of certain inclusion criteria (e.g. type of surgery). However, to minimize potential selection bias, we will analyze the data stratified by type of surgery and explore the interaction between the type of surgery and the randomization group (intervention vs control). Finally, since the study is conducted in a single hospital, there is the potential of limited generalizability of the findings to broader patient populations. Nonetheless, the study is conducted at the largest hospital in Catalonia, with a potential population reach of more than 430000 inhabitants, being the reference for these surgeries in the entire region.

### Current status of the RCT

Patient recruitment began in May 2023 and currently 25 subjects have been recruited. The pace of recruitment is slow because many eligible patients decline the invitation. In view of this scenario, since the beginning of the RCT a database has been created with details of the eligible participants who refuse to participate and their reasons for use in subsequent analysis.

The RCT follows the plan of the proposed intervention closely and we hope to reach the sample size necessary, albeit more slowly than expected.

## CONCLUSIONS

If the present study demonstrates the efficacy of this opportunistic intensive intervention in smokers about to undergo surgery, it seems justified to propose its implementation in the standard clinical practices of the rest of the surgical specialties of the Catalan Health System.

## Supplementary Material



## Data Availability

Data sharing is not applicable to this article as no new data was created.
